# Implementation of community based advance distribution of misoprostol in Himachal Pradesh (India): lessons and way forward

**DOI:** 10.1186/s12884-018-2036-2

**Published:** 2018-10-29

**Authors:** Rakesh Parashar, Anadi Gupt, Devina Bajpayee, Anil Gupta, Rohan Thakur, Ankur Sangwan, Anuradha Sharma, Deshraj Sharma, Sachin Gupta, Dinesh Baswal, Gunjan Taneja, Rajeev Gera

**Affiliations:** 1 Health Systems, USAID-VRIDDHI/IPE Global, New Delhi, India; 2 Maternal Health, Department of Health and Family Welfare, Government of Himachal Pradesh, Shimla, India; 3 Maternal and Newborn Health, USAID-VRIDDHI/IPE Global, New Delhi, India; 4 USAID-VRIDDHI/IPE Global, Shimla, Himachal Pradesh India; 5 USAID-VRIDDHI/IPE Global, Mandi, Himachal Pradesh India; 6 USAID-VRIDDHI/IPE Global, Kinnaur, Himachal Pradesh India; 7 Department of Health and Family Welfare, Government of Himachal Pradesh, Mandi, Himachal Pradesh India; 8 Maternal and Child Health, USAID-India, New Delhi, India; 9 Maternal Health, Ministry of Health and Family Welfare, Government of India, New Delhi, India; 10 USAID-VRIDDHI/IPE Global, New Delhi, India

**Keywords:** Maternal mortality, Misoprostol, Post-partum hemorrhage

## Abstract

**Background:**

Postpartum Hemorrhage remains the leading cause of maternal mortality. To prevent PPH, Misoprostol tablet in a dose of 600 micrograms is recommended for use immediately after childbirth in home deliveries wherein the use of oxytocin is difficult. The current article describes an implementation of “community based advance distribution of Misoprostol program” in India which aimed to design an operational framework for implementing this program.

**Methods:**

The intervention was carried out in Janjheli block in Mandi district of the state of Himachal Pradesh which is a mountainous terrain with limited geographical access and reported 90% home deliveries in the year 2014–15. An operational framework to implement program activities was designed which was based on WHO HSS building blocks. Key implementing steps included- Ensuring local ownership through program leadership, forecasting and procurement of 600 mcg misoprostol tablets, training, branding and communication, community engagement and counselling, recording and reporting, monitoring, supportive supervision and feedback mechanisms.

**Results:**

Over the one year of implementation, 512 home deliveries were reported, out of which 89% received the tablets and 84% consumed the tablet within one minute of delivery. No incidence of PPH in tablet consuming mothers was reported. On account of periodic counselling and effective community engagement the intervention also contributed to better tracking of pregnancies till delivery and institutional delivery rates which increased to 93% from 45% and 57% from 11% respectively as compared to the preceding year.

**Conclusions:**

The model has successfully shown the use of single misoprostol tablets of 600 mcg, first time in this program. We also demonstrated a HSS based operational framework, based on which the program is being scaled to additional blocks in Himachal Pradesh as well as to other states of India.

**Electronic supplementary material:**

The online version of this article (10.1186/s12884-018-2036-2) contains supplementary material, which is available to authorized users.

## Background

Maternal mortality remains a key concern across the globe with maternal deaths being a core marker of the functioning of the public health care delivery system. Currently 52% of the maternal deaths (in pregnancy, at or soon after childbirth) globally are attributed to the three leading preventable causes of hemorrhage, sepsis and hypertensive disorders [1]. Based on global data from 2003 to 2009, maximum maternal deaths were attributed to hemorrhage and it accounted for more than 27% of maternal deaths in the developing regions and approximately 16% in the developed regions [2]. With a Maternal Mortality Ratio (MMR) of 167 per 100,000 in 2013, India accounts for a substantial number of maternal deaths worldwide [3]. Also, in line with the global scenario hemorrhage accounts for 40% maternal deaths in India with Post-Partum Hemorrhage (PPH) being the major contributor [4].

Recognizing this important fact, the use of Uterotonics immediately after birth has been universally advocated to prevent the occurrence of PPH [5]. Among the available Uterotonics, oxytocin remains the drug of choice with misoprostol identified as the alternate. However a major concern for prevention of PPH is that the administration of oxytocin, requires the assistance of a skilled birth attendant (SBA), and therefore is not available to women experiencing unattended home births, either by choice, lack of access to SBAs [6, 7] or due to gender and wealth disparities [8–10].To address these concerns as regards uterotonic cover, the use of misoprostol for the prevention of PPH by community health care workers and lay health workers is recommended in settings where skilled birth attendants are not present [5]. Various studies have demonstrated misoprostol’s effectiveness in preventing PPH, reducing the need for additional interventions and minimizing the need for referrals in a variety of community-based settings [11]. The World Health Organization (WHO) guidelines on “Optimizing health worker roles to improve access to key maternal and newborn health interventions through task shifting” also advocates for the distribution of misoprostol by lay health workers for home births [12]. Multiple programs across the globe have been implemented using misoprostol for prevention of PPH in home births using different approaches [13] and it is now widely recognized that distribution of misoprostol through community health workers (CHWs) for PPH prevention could be a significant step in reducing maternal deaths in low-resource settings [14].

Though the institutional delivery rate in India has improved considerably with the National Family Health Survey (NFHS) 4 data reporting it as 78.9% in 2015–16, an increase of 40.2% as compared to NFHS 3 (2005–06) [15], the country still has geographical pockets with high home delivery rates. As pointed out by global evidence that occurrence of PPH remains high in home deliveries and keeping in perspective the use of misoprostol in home births, the Ministry of Health and Family Welfare (MoHFW) in India undertook the policy decision to permit Accredited Social Health Activists (ASHAs) to undertake advance distribution of misoprostol to pregnant women who are likely to deliver at home to prevent PPH in 2013. Within the Indian context, ASHA is identified as a community health volunteer who creates awareness on health and its social determinants and mobilizes the community towards local health planning and increased utilization and accountability of the existing health services.

This is a marked shift from the initial decision of only allowing Auxiliary Nurse Midwives (ANMs) to administer misoprostol during home deliveries and is supported by global evidence on the effectiveness, feasibility and safety of advance distribution of misoprostol to pregnant women to prevent PPH [13, 14, 16]. Positive results from other low resource setting countries like Bangladesh, Nepal and Zambia which have implemented and scaled up misoprostol for prevention of PPH were also instrumental for Government of India in moving forward with implementing this intervention in the country [17].

### Rationale for the intervention

**Fig. 1:**
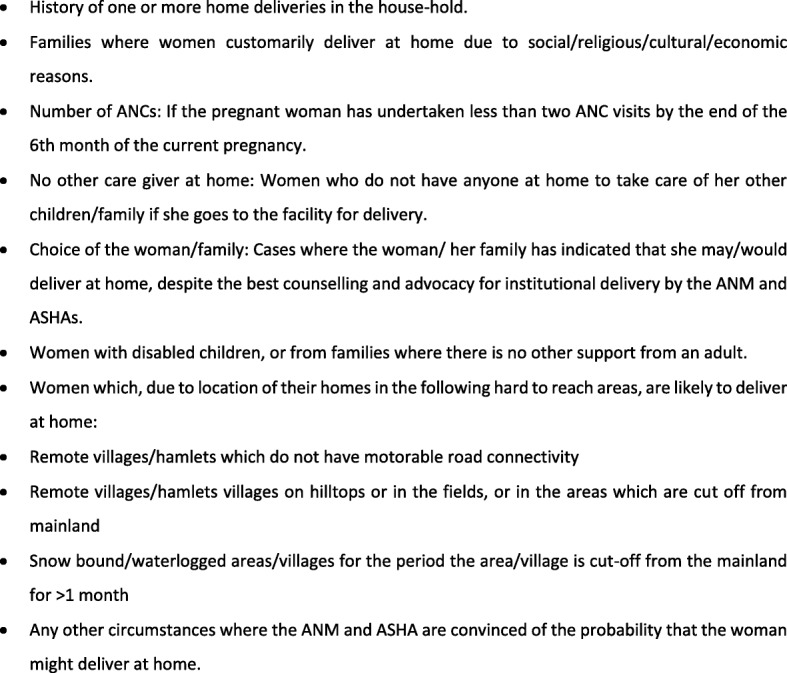
Criteria to identify a pregnant woman with likelihood of home delivery (based on GOI guidelines)

In line with global evidence, MoHFW, advocates for advance community-based distribution of misoprostol to pregnant women on completion of the 8th month of pregnancy by ASHAs in identified high home delivery geographical pockets to women who are likely to deliver at home [4]. The national guidelines put forward criteria to help CHWs to identify women who have a greater chance of delivering at home and hence need to be provided the misoprostol tablets [4]. (Fig. 1):

Though the intervention is an exception to the policy promoting institutional deliveries, it has been enlisted as one of the major interventions within the 5 × 5 matrix developed by Government of India (GOI), which prioritizes high impact interventions across the RMNCH+A spectrum [18].However, so far the uptake of the program has been low with no state in the country having implemented and scaled up the program despite the availability of national guidelines. Two main reasons cited for the intervention not taking off in the country were apprehensions towards offsetting the efforts of promoting institutional deliveries and a leakage of the drug in the system with its possible misuse. In addition, other factors such as dependency of the programme activities on a CHW, drug related adverse events and the operational challenges in implementing the intervention in far off low resource settings requiring strict monitoring and tracking were also cited to be responsible for poor offtake.

To reiterate the importance of the intervention MoHFW reconvened a National Level Workshop in February 2016 for selected states with identified pockets of home deliveries. The workshop brought forward evidences of fractured implementation across the states of Chhattisgarh, Jharkhand and Madhya Pradesh. A need was therefore felt by the national health ministry to identify a framework which could pave the way for effective implementation across the country.

## Methods

### Aims of the intervention

The current intervention aimed to design and implement an operational framework to implement and scale up the “Community Based Advance Distribution of Misoprostol” program in India.

### Objectives

#### Primary objectives

- To improve uterotonic coverage in the intervention area by advance distribution of misoprostol tablets by community health workers for prevention of PPH in home births
- To test the operational framework for the intervention and understand determinants for success of implementing a community based program in remote, disconnected and a very low resource setting in the Indian context

#### Secondary objectives

- To understand and report on any other emerging systemic effects of the intervention including an impact on institutional deliveries

### Implementation design

The intervention has been designed and implemented based on the WHO Health System Strengthening (HSS) building blocks keeping fidelity with the national framework [4] using existing Antenatal Care (ANC) platforms and CHWs in the public health care delivery system.

The results and achievements have been measured for the following key variables:

- Uterotonic coverage
- Pattern of drug distribution and consumption
- Occurrence of PPH in beneficiaries who consumed the tablet (The operational definition for identification of PPH was used as stated in the national guidelines - a blood loss of more than 500 ml or soaking of an average sized pad in 5 min) [4]
- Drug related adverse events

In addition, the intervention also studied the influence on health system in the intervention geography including tracking of pregnancy outcomes and institutional deliveries through a mix of qualitative and quantitative approaches.

#### The implementation geography

The state of Himachal Pradesh is a predominantly mountainous state set in the lap of Himalayas in north-west India with an altitude range from 350 m to 6975 m. Climatic conditions vary with heavy snowfall in winter months in higher altitudes making many areas inaccessible [19]. Hence home delivery rates are particularly higher in rural areas in hilly terrain which are geographically difficult to access and have traditionally been pockets of home deliveries. Contextualizing the geographical and climatic conditions the state offers apt conditions to implement the “Advance Community Based Distribution of Misoprostol” program.

It was decided to pilot the implementation in one particular geography with subsequent scale up in other regions of the state. The USAID funded VRIDDHI (Scaling up RMNCH+A Interventions) Project has been providing technical assistance to the state government across the four High Priority Districts (HPDs) of Chamba, Kinnaur, Lahaul-Spiti and Mandi as part of the RMNCH+A strategy in India. Within these districts, high home delivery rates and geographical access were the factors on which the implementation geography was identified. A review of Health Management Information System (HMIS) data for the year 2015–16, identified the community development block of Janjheli in Mandi district with the highest home delivery rates and hence the same was identified for piloting the intervention.

Set at an altitude of 2150 m above sea level in the western Himalayan range, Janjheli has a population of 86,597 (93.73% rural) with two distinct population clusters located at a distance of three to four hours from the district headquarters. The block has difficult terrain with large portions cut off due to snowfall in the winter months. It has 33 public health facilities including one Community Health Center (CHC), 7 Primary Health Centers (PHCs) and 22 Health Sub Centers (HSCs) with only the CHC functioning as a delivery point and providing Basic Emergency Obstetric Services (BEmOC). Access to a Comprehensive Emergency Obstetric Services (CEmOC) center is a major challenge with the nearest one being 3–4 h away from the BEmOC center. The block has a shortage of Medical Officers with 11 medical officers posted at the CHCs and PHCs against a sanctioned strength of 14. Hence the Frontline Health Workers (FLWs) including the 2 health supervisors, 25 Auxiliary Nurse Midwives (ANMs) and 103 Accredited Social Health Activists (ASHAs) form the primary source of health care service delivery beyond CHCs and PHCs.

The implementation began in April 2016 and data from April 2015 – March 2016 was reviewed to assess the Antenatal Care (ANC) and delivery rates. The data review stated that though against an estimated annual cohort of 1700 pregnant women, 1490 women were registered for ANC with 902 (60%) completing three ANC checkups, for the corresponding period only 667 deliveries were reported with 596 (89%) being home deliveries. This is reflective of the Health system not being able to track all registered pregnant women and their delivery outcomes.

#### Intervention population

##### Inclusion criteria

The beneficiaries of the program were included from all villages of Janjheli block as follows:

- All Pregnant women along with their family members for purpose of counselling (estimated 1400 pregnant women in one year)
- Any visiting pregnant women coming in contact with Front Line Workers (FLWs) during the period of implementation of program

##### Exclusion criteria-

- Pregnant women and families falling outside administrative area of Janjheli and not visiting Janjheli area during the pregnancy period

The program personnel involved in the implementation included the following -

- All FLWs of the block – 6 health supervisors, 26 health workers /ANMs and 103 ASHAs

All health department officials of the block i.e. Block Medical Officer (BMO), block pharmacist, medical officers (MO) at PHCs in the block.

#### Operational framework -

##### Program implementation using the WHO HSS based operational framework [20]–

Moving forward on the recommendations from the National Workshop of February 2016, the state of Himachal Pradesh commissioned the program on “Community Based Advance Distribution of Misoprostol tablets to prevent PPH” through the state National Health Mission (NHM). The entire implementation design was developed using the national guidelines. However certain adaptations were undertaken to contextualize the intervention to local needs. This included adaptations for the training material, job-aids, methodology of training and monitoring of the implementation.

The VRIDDHI team worked with the state government and provided technical support for developing the operational framework and its roll-out. Being a government approved program, no ethical clearance was sought prior to initiating the implementation. The state government and the VRIDDHI team undertook meticulous planning prior to the actual roll out of the program.

Key activities undertaken during the implementation phase include the following:

#### Leadership and governance

A state level steering committee was established under the leadership of the Mission Director (MD), NHM to guide and facilitate the implementation process. The Program Officer (Maternal Health) was the designated nodal officer at the state and district levels while the local level leadership was provided by the block Medical Officer (MO).

#### Health products - procurement and packaging of tablet

As per GoI guidelines misoprostol is to be administered in the dose strength of 600 mcg to the mother post-delivery for prevention of PPH. However, a major challenge existing in the implementation process was unavailability of the drug in the required strength within the public healthcare system in India. Till date three tablets of 200 mcg were being used for consumption by the beneficiary which made packaging difficult. Availability of only a single vendor manufacturing the 600 mcg tablet further compounded the situation. The state through a major policy change in the procurement process approved single vendor procurement for the 600 mcg drug at the district level. This decision not only facilitated the current program implementation but has positive ramifications on the overall procurement related policies and processes in Himachal Pradesh.

**Fig. 2:**
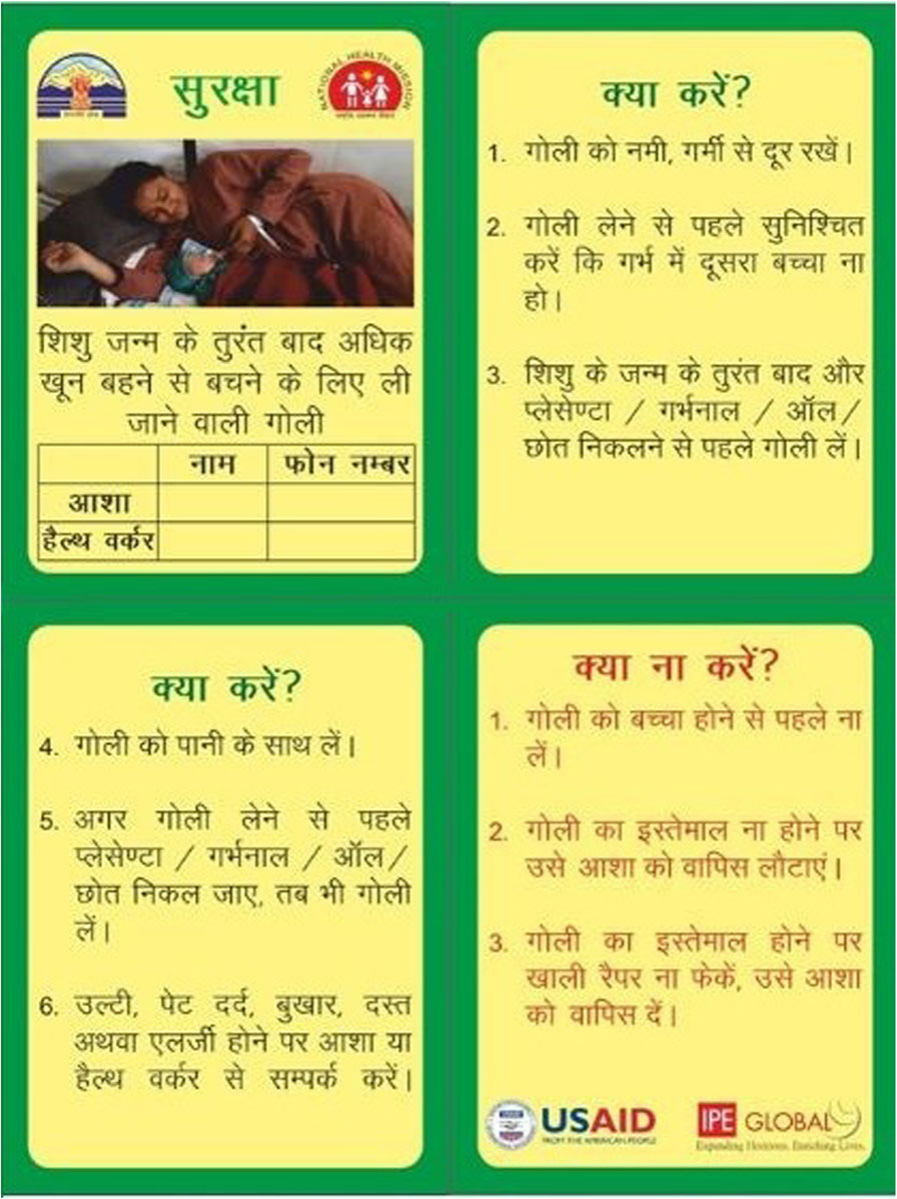
Pack insert: highlighting Do’s and Don’ts for misoprostol use

The drug was delivered to the beneficiaries with a guidance leaflet inserted in the drug pack which clearly elaborated key messages regarding the tablet (Fig. 2). In addition to facilitate tracking of distributed, consumed and returned tablets a unique identification number was given to each dose being distributed in the community.

A major initiative was branding of the intervention as “SURAKSHA” which essentially means “Safety” to convey the importance of the drug to the beneficiaries. The drug packets, training and communication tools being used during the implementation phase were branded with the same terminology to convey a homogenous message to all concerned stakeholders.

#### Human resources - development of training and communication material

**Fig. 3:**
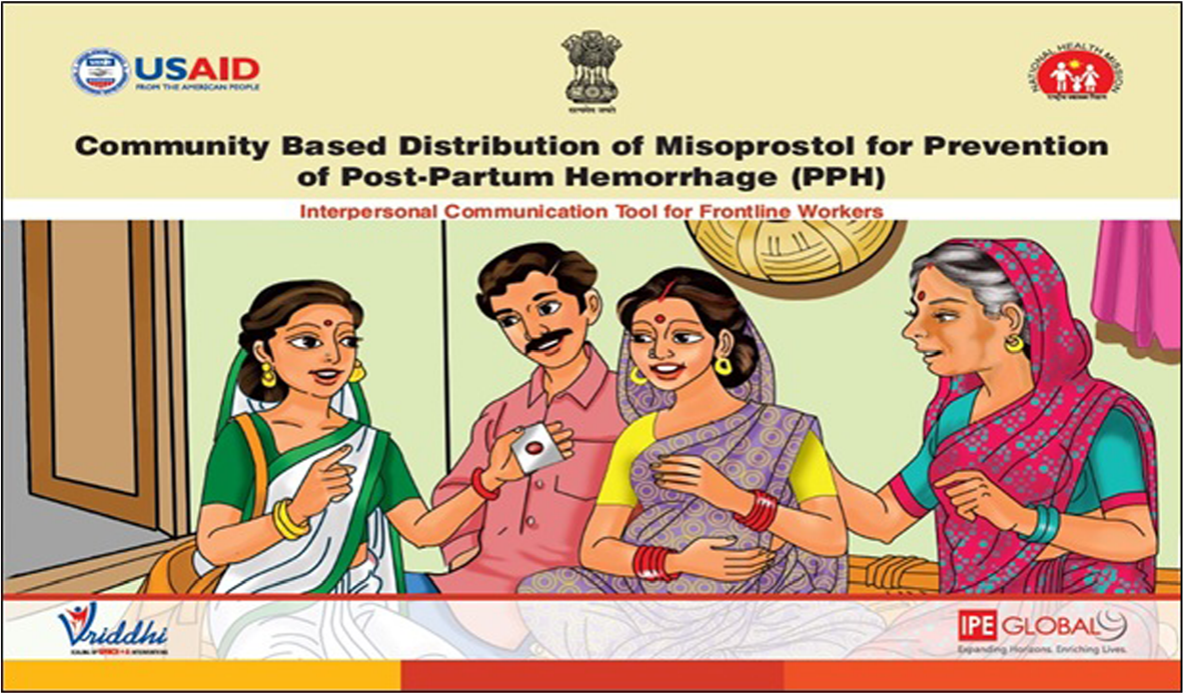
The Inter-Personal Communication flipbook designed for the intervention

Based on the national guidelines due emphasis was given to create a structured and comprehensive training package for the FLWs and communication tools were developed and translated in local language to aid in program service delivery. The national guidelines were adapted into local language and a program booklet containing the program description, roles and responsibilities of service providers was developed for the FLWs. A flip book incorporating all key messages was developed as an Interpersonal Communication and Counselling Tool (IPCC) for the ASHAs for their interaction with expectant mothers (Fig. 3).

The intervention was initiated with a one-day district level orientation on the program guidelines for the district level health officials and program managers in April 2016. This was followed by two block level trainings for the FLWs wherein a total of 100 ASHAs (CHWs), 23 ANMs, block pharmacist and Medical Officer in Charge (MOIC) were trained on the intervention using the program flip book as the training tool. Participatory training methodology was used for the training which provided an opportunity to discuss and deliberate on all issues of the program intervention with the service providers. Key training content included information on the dosage, timing (within a minute of delivery) and route of drug administration and side effects related to the drug. Special impetus was on stressing upon the importance of institutional deliveries which also formed the central theme during the subsequent counselling sessions between the ASHAs and beneficiaries during the drug distribution on completion of the 8th month of pregnancy. In addition, the distribution of tablets to the FLWs was also undertaken during the training, based on the delivery load for the subsequent quarter in the coverage area. The ANMs of the respective HSCs were involved as field depots to supplement the ASHA with tablets if required.

#### Health service delivery

##### Tablet distribution process –

To distribute the tablets, AHSAs started visiting the households of pregnant women who had completed their 8th month of pregnancy from the next day of their training. Households were identified from the line list of pregnant women which were made by CHWs for their areas before the trainings.

**Fig. 4:**
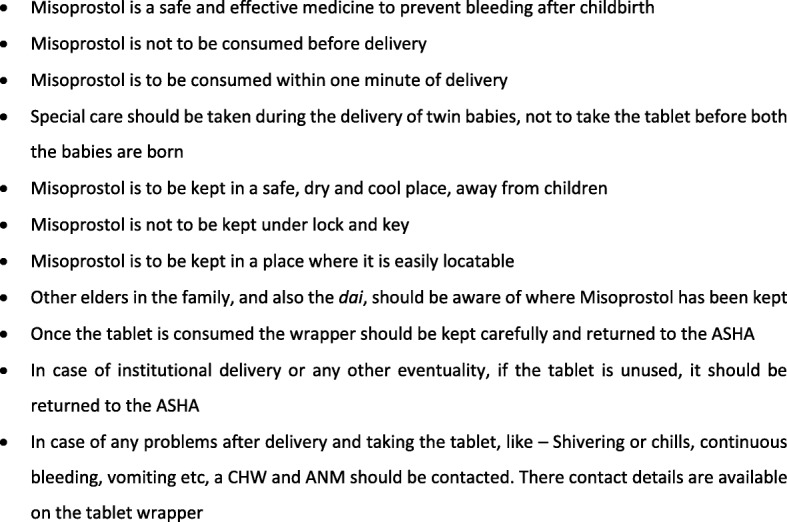
Key messages explained by the ASHAs and ANMs to the pregnant women provided with Misoprostol

The distribution work of tablets and related counselling of families was inducted as an additional work for the CHWs in their routine. However, visiting households of potential beneficiaries for reproductive, maternal and child health services was part of exiting routine of the CHWs. This included visiting married couples for family planning services, visiting pregnant women to mobilize them for ante natal checkups and for visiting health facilities for delivering babies, post-delivery follow up care to mother and home-based care for newborn babies. The introduction of ‘misoprostol’ tablet distribution by CHWs did fit well with the antenatal care support to these PWs and was used as an opportunity to create an additional contact between CHWs and PW or their family members. A detailed, structured counselling process with tablet distribution anchored the message around importance of going to a health institution for delivery**.** (Fig. 4).

#### Health information systems

##### Recording and reporting –

The recording formats as available in the national guidelines were adapted and improvised to create a comprehensive recording and reporting mechanism after taking in inputs from involved stakeholders at all levels. The intervention was initiated with a line listing of current pregnancies in the geography and an Excel based Management Information System (MIS) tool was developed to maintain a database of the beneficiaries as regards consumption of misoprostol tablets and tracking of each drug distributed by collection of unused drug/ wrapper in case of used drug (Additional file 1).

##### Supportive supervision, monitoring and review mechanisms –

A supportive supervision mechanism was institutionalized within the intervention with a structured community monitoring tool developed to facilitate the process. Multiple supportive supervision visits were undertaken primarily by involving the first line supervisors (Lady Health Visitors), block and district level officials and VRIDDHI project team staff. The post-delivery follow up visit served as a method to track the beneficiaries for their pregnancy outcomes, consumption of tablet and any adverse events experienced. Cluster and block level review meetings were held every month to review the progress, identify challenges and refine the intervention. The block level meetings were also used as a platform to assess the tablet usage and distribute tablets to ASHAs as per estimated need.

#### Health financing

##### Financial incentives –

The distribution of tablet misoprostol at the community level is dependent on ASHA who is a community linked volunteer and incentivized for each service provided. Therefore, a financial incentive structure in line with other health programs has been incorporated for them in the program intervention. The ASHAs were paid a financial incentive of INR 200 for every tablet consumed and INR 100 for return of unused tablet. Moving forward the state has also requested the national ministry for a fixed monthly incentive of INR 300 for submission of monthly reports to the reporting units.

## Results

**Fig. 5:**
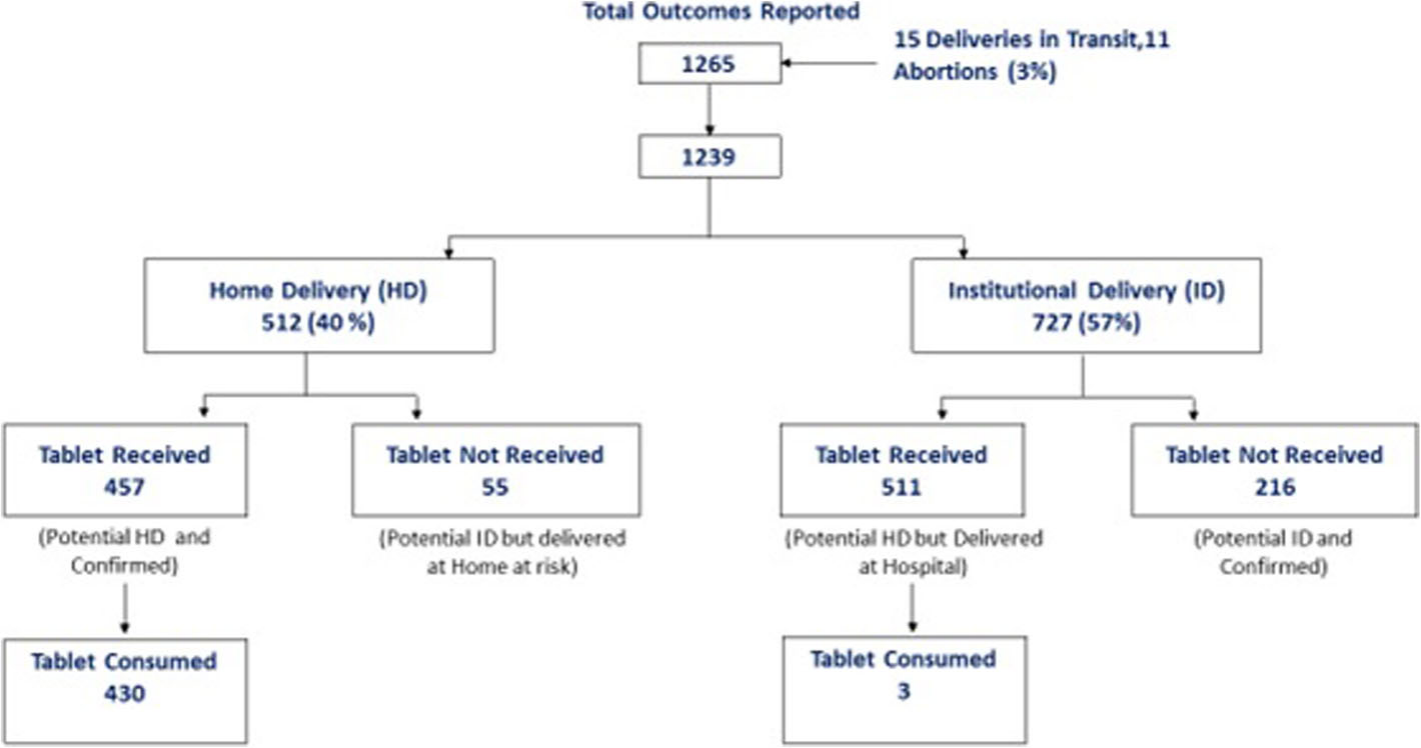
Tablets distributed and consumed during the intervention

A total of 1265 pregnancy outcomes were reported in Janjheli from 5th May 2016 to 31st May 2017, against 1422 antenatal registrations. Out of this 512 (40%) were reported to be home deliveries, 15 (1%) women delivered on the way to a health facility and 727 (57%) delivered in health facilities, while 11 loss of pregnancy (abortions) were reported. Of the total 1265 deliveries reported, misoprostol was distributed to 979 women who were identified for possible home deliveries. Of the 979 women who were distributed the tablet 511 (52%) had an institutional delivery, 11 (1%) delivered on transit and 457 (47%) had a home delivery. Of the 512 home deliveries reported during the intervention period, the drug was distributed to 457 (89%) women and consumed by 430 beneficiaries thus achieving an uterotonic coverage of 84% for all home deliveries. 55 (11%) women who delivered at home were not distributed the tablet as they were not pre-identified in the home delivery group while 27 (6%) women who were distributed the drug, did not consume the tablet. 6 of these women misplaced the tablets, 7 delivered at their maternal place and did not carry the tablet with them while 11 did not consume it because of apprehensions related to the use (Fig. 5) (Additional file 1).

419 (98%) out of 430 tablets were consumed within 1 min or immediately after delivery of baby while none of the women consumed the tablet before the delivery of the newborn, as reported from the follow up visits of the community health workers.

Three women who had institutional deliveries but carried tablets with them consumed the tablet at the health facility itself, while 8 women who delivered on the way while reaching a health facility consumed the tablet. Of the 979 doses distributed, 932 (96%) were successfully tracked either through returned wrappers or unused drugs to the AHSA workers during visits undertaken by them during the Post Natal period. Overall no incidence of PPH was reported among women who consumed misoprostol and only 14 minor adverse events were reported during the intervention period (Table 1). In addition, no maternal death was reported from Janjheli in this period.

**Table 1:** Adverse events reported following Misoprostol use

Adverse events reported (*N* = 430 women who consumed Misoprostol tablet after home based delivery)	Number
Mild Fever	4
Nausea	2
Vomiting	1
Diarrhoea	2
Pain Abdomen	1
Mild tremors	2
Vertigo	1
Temporary Ptosis	1

An increased involvement of the community with the health system resulted in improved trust between the two. The interaction between the ASHAs and the pregnant women on completion of the 8th month close to the Expected Date of Delivery (EDD) resulted in a fivefold increase in the institutional delivery rates which increased to 57% from just 11% (71 of the 667 reported deliveries) in the preceding year (April 2015 – March 2016). In addition, the interaction also potentiated tracking of pregnancies till delivery. While from April 2015 – March 2016, only 667 (45%) total deliveries were reported against an ANC coverage of 1490, during the intervention period 1265 (89%) pregnancy outcomes were known against 1422 antenatal registrations.

Besides having a direct impact on improving the uterotonic coverage in the home deliveries in the area, the intervention resulted in overall Health System Strengthening (HSS) in Janjheli block. Enhanced capacity of healthcare providers for service delivery, institutionalizing the supportive supervision system, strengthened reporting and effective use of data, improved feedback through regular cluster and block level review meetings and demand generation through community engagement and ownership were key additional learnings from the intervention.

The tangible outcomes of health system improvement as evident from the results of this study were – better tracking of pregnancy outcomes and improved institutional delivery rates. The positive effect on various aspects of the health systems in Janjheli block were realized by discussions with the block health functionaries and observations made during the process of implementation. The block health officers recognized that the monthly meetings at block level and cluster level have become more regular and a positive feedback loop during these meetings has become a part of review of other health programs as well. Similarly, the recording and reporting tools used for this intervention are also helping implementation of other programs as they have allowed community health workers to have systematic records of all the pregnant women of their area. The interaction with the community health workers potentiated the fact that the current intervention has built their capacities to communicate with the beneficiary families and also engage them to utilize available health services better. This is also more relevant in the context of Janjheli block, as the community health workers (ASHAs) were commissioned only few months before the intervention and had only gone through an orientation before the trainings for this program were conducted.

**Fig. 6:**
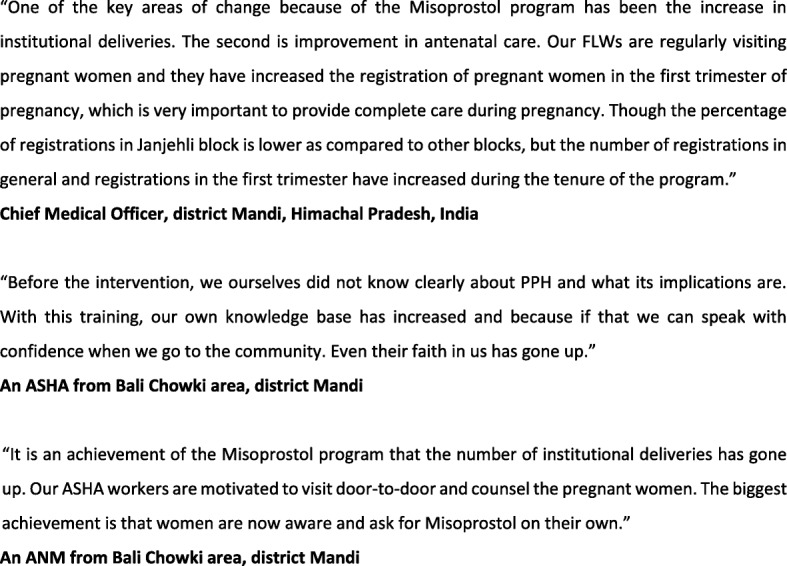
Experience of Field staff

There was a positive shift perceived in the antenatal care experience of PW as identified by the CHWs and through follow up interviews by the program supervisors and documentation teams. This could be attributed to an additional antenatal contact at households and a counselling process which allowed enough time to be spent at the households by CHWs. While we did not capture any additional data to look at improved ANC services coverage rate, we interviewed the benefitted family members and CHWs on their experience. Some of the voices which emerged during experience sharing visits are quoted below in Fig. 6.

## Discussion

Community based advance distribution of misoprostol tablets for prevention of PPH in home deliveries has been globally recognized as an important intervention contributing to ending preventable maternal deaths [5]. The current intervention has successfully provided a model for effective implementation of the program in India with important implications in reducing incidence of PPH and maternal mortality during home deliveries in the country. Though the country has substantially increased institutional delivery rates, still 6.3 million deliveries are conducted at home. At current national MMR, within this cohort of home deliveries 10,571 maternal deaths are expected with 4228 (40%) being contributed by PPH [4]. Scientific data states that atonic causes contribute to 90% of PPH cases [21–23] and this single intervention reduces deaths due to PPH in home deliveries by 50% [24]. If implemented at 100% coverage the intervention has a potential to possibly avert 2114 maternal deaths across the country with more conservative estimates at 70% coverage would avert 1480 maternal deaths across India with an overall reduction in maternal deaths by 3.4–4.8%. Rigorous modelling exercises extrapolated for population estimates in Bangladesh have also demonstrated a reduction of incidence and risk due to death from PPH in home deliveries [25].

The overall uterotonic coverage rate achieved in the current intervention was 84%, which is in line with findings from earlier studies [26–29]. In line with previous studies which have reported minor and transient side effects [26, 30], the current intervention also reported 14 minor, self-limiting side effects (4%) and no PPH case among the 351 women who consumed misoprostol thus strengthening evidence for scale up strategies in India.

The intervention has been able to offset the apprehensions regarding a negative effect on institutional deliveries and drug leakage and misuse. The institutional delivery rate in the area increased five times from 11 to 57% within 6 months of implementation possibly because of an additional contact between the ASHA and the beneficiaries close to the date of delivery and stressing upon the importance of institutional delivery during the counselling sessions. While there is a potential contribution of better tracking of deliveries to the increased proportion of institution-based births, this finding is also in line with three other global interventions which reported an increase in institutional delivery rates during their intervention periods [31–33]. As with the current intervention, the three other programs also appeared to put a high value on counselling of the woman and her family regarding the importance of skilled attendance at birth, the dangers of PPH, and the use of misoprostol only for the situation where a woman is unable to achieve her plan of a facility-based birth [13].

96% of the distributed drugs were successfully tracked in the intervention with this rate being similar to a study in Ghana in which 98.3% of the doses were successfully tracked and leaks into the system prevented [34]. Giving each tablet a unique identification number and institutionalizing comprehensive operational components of supportive supervision and review meetings were possible reasons for this and again will be defining strategies for scaling up the intervention.

In line with other studies with similar distribution models 98% of the beneficiaries (419 out of 430 home deliveries who received the tablets) consumed the drug within a minute of delivery and as intended no incidence of consumption of the drug was reported prior to delivery, thus underlining the effectiveness of the implementation framework [31, 32, 34].

The current intervention is possibly the first one wherein a single 600 mcg misoprostol has been used instead of the three tablets of 200 mcg each in the public health care system. Though procurement of a single dose 600 mcg tablet was difficult, the state government through pro-active policy level decision making was able to procure the single dose tablet at district level. This not only eased the packaging and distribution of tablet but additionally may have had a positive impact on beneficiary compliance because of ease of consumption of one tablet instead of the usual three, though evidence for the same is currently lacking [35]. Often poor procurement policies limit the success of public health initiatives, but the same was overcome in this program. Taking learnings from the current initiative other states in the country are now procuring the 600 mcg tablet for distribution. Inclusion of the 600 mcg tablet in the country’s Essential Drug List (EDL) at all levels of health care will further simplify the process which currently includes only the 100 and 200 mcg tablets for use at tertiary level health facilities [36].

Supply Chain management principles were applied to ensure regular supplies and sufficient stock of tablet Misoprostol. The unique identification number given to each tablet contributed towards effective tracking of the tablet.

Capacity enhancement of the service providers was undertaken through multiple measures during the implementation. A well-designed interactive training package helped improve the understanding and competency of the health workers towards the program while supportive supervision visits and review meetings further potentiated their efforts for effective service delivery. The line listing of pregnant women undertaken at the start of the implementation and the subsequent tracking of beneficiaries helped the service providers have a better understanding of their work area.

Close coordination and team work among the different cadres of health officials and functionaries was demonstrated during the intervention. While the state and district level officials provided clear cut policy level support and guidance, the block MOIC and his team of ANMs, ASHAs, program manager and pharmacist at CHC Janjheli worked closely to ensure smooth and effective service delivery.

Programmatic interpretation and adaptation of the national policy also contributed towards the results. For example, on data review after 4 months of implementation, it was realized that many women having home deliveries were being missed out as they were earlier identified to likely deliver at an institution. Post review the FLWs were advised to ascertain the likelihood of home delivery in PW taking into account the access and time to care and distribute misoprostol to pregnant women who despite all intentions would not be likely to access an institution for delivery. This decision for a more liberal distribution of the drug was taken at the start of winter months with stress on ensuring tracking and collection of unused drug / wrapper and proved pivotal in improving coverage in the later months.

The intervention has again reiterated the importance of active community engagement as a critical contributing factor for the success of any health intervention. Counselling sessions focused at the beneficiary and family members and the contacts between the service providers and the beneficiaries close to the date of delivery not only improved the compliance for institutional deliveries but also improved the trust factor between the two. This aspect of the intervention can definitely help facilitate the implementation of other health programs in the geography.

It is thus imperative that implementation of a community-based program in remote, low resource geographies has to negotiate through challenges of existing program implementation practices. There is a likelihood of unclear program processes clubbed with lack of human resources and their capacities for carrying out these processes. Hence, designing and executing good capacity building measures, carefully curated processes which are co-designed with all involved stakeholders, sustained handholding and problem-solving support, building easy to use data flow processes and relevant mid-course corrections are of high importance.

### Limitations of the study –

The current intervention has some limitations in the sense that all the data was reported by the service providers and is subject to bias. However, the tracking of wrappers can be considered as a proxy to the validity of the data. Though the current intervention did not report any incidence of PPH in beneficiaries who consumed misoprostol the relative difficulty in measuring PPH in community based setting can be considered as a limitation. In addition, being a proven intervention with demonstrated effectiveness no baseline and end-line surveys to compare the incidence of PPH was conducted in the intervention area and the entire focus was on optimizing the implementation of the intervention.

## Conclusion

Advanced community based distribution of misoprostol has been effective across multiple countries and the current intervention is the first one to demonstrate effectiveness of the program in a field setting in India. The model has successfully demonstrated the implementation of the GOI framework and is now not only being scaled to 11 additional blocks with high home deliveries in the state of Himachal Pradesh, but also to other states of the country. Being a community based intervention, it provides opportunities to build upon other programs targeted at the same geography. Facilitation strategies such as flip book with all key counselling messages, pamphlet with messages on do’s & don’ts to beneficiary and documented roles and responsibilities of key stakeholders helped in maintaining implementation fidelity to government programme. The success of this community intervention was also enabled due to constant support and handholding of the FLW delivering the implementation through regular supportive supervision and review by district and state level officials. The intervention also negated the assumption of offsetting the trends of institutional deliveries and in fact contributed to a fivefold increase in hospital based deliveries on account of additional contacts between the service providers and the beneficiaries which can have huge implications in reframing of the Ante-Natal Care program in the country.

Key recommendations from the intervention include the use of single 600 mcg tablets instead of the 200 mcg tablets, inclusion of a financial incentive package for the ASHA workers and a more liberal distribution of the tablet to all pregnant women in the intervention geography instead of identifying women with higher chances of home deliveries. Successful scale up of this intervention can contribute towards significant reduction in maternal deaths in the country thereby help achieve the SDG targets.

## Additional file

Additional file 1: Monthly Data base and Program Indicators. The data file attached provides a complete data set used for this pilot implementation. The data file includes the following sheets – Sheet 1 - For monthly data – One sheet for each monthly data set for the block from May 2016 to May 2017 – these sheets include sub health center wise details of expected and reported deliveries (at home and at institutions), tablets distributed, tablets consumed, tablets and wrappers collected back, adverse events reported, complications reported including PPH cases and any referrals **(All these sheets are named month wise for example – May_16, Jun_16 and so on till May_17).** Sheet 2 - For cumulative numbers for all parameters in monthly data sheets (Named as– **‘Cumulative data’).** Sheet 3- for sub health center wise cumulative data **(Named as– ‘Subcenter Wise’)**. Sheet 4 - A block level Cumulative Summary of main measurement indicators for the program for total duration of pilot implementation. This sheet includes – expected and reported outcomes of pregnancy in the block, numbers of tablets distributed and consumed, number of tablets collected back and PPH cases reported. All of these numbers are further divided according to place of reported deliveries at home, at institution and in transit. **(Named as – ‘Program Indictors Cumulative’).** Sheet 5 - Various charts showcasing trends of data. **(Named as – ‘Charts’). (XLSX 222 kb)**

## Abbreviations

ANC: Ante Natal Care
ANMs: Auxiliary Nurse Midwives
ASHAs: Accredited Social Health Activists
BEmOC: Basic Emergency Obstetric Services
BMO: Block Medical Officer
CEmOC: Comprehensive Emergency Obstetric Services
CHC: Community Health Center
CHWs: Community Health Workers
EAG: Empowered Action Group
FLWs: Frontline Health Workers
HMIS: Health Management Information System
HPDs: High Priority Districts
HS: Health Supervisor
HSCs: Health Sub Centers
HSS: Health System Strengthening
MD: Mission Director
MDGs: Millennium Development Goals
MMR: Maternal Mortality Ratio
MO: Medical Officers
MOIC: Medical Officer in Charge
NFHS: National Family Health Survey
PHCs: Primary Health Centers
RMNCH+A: Reproductive Maternal Newborn Child and Adolescent Health
SBA: Skilled Birth Attendant
SDGs: Sustainable Development Goals
SOPs: Standard Operating Procedures
WHO: World Health Organization
